# 

**Published:** 1902-05-24

**Authors:** 


### NOTICE TO CORRESPONDENTS.

All MSS., Letters, Books for Review, and other matters intended for the Editor should be addressed The Editor. "The Hospital," The Hospital Building, 28 & 29 Southampton Street, Strand, W.C.
The Editor cannot undertake to return rejected MS., even when accompanied by stamped directed envelope.

**Notice to Advertisers and Subscribers.**
All Advertisements, Orders for Copies of The Hospital, and Business Communications should be addressed to **THE MANAGER, (Not to the Editor)**, The Hospital, 28 & 29 Southampton Street, Strand, London, W.C.
The Cost of Subscription, post free, is as follows.—Per week, 2½d.; per quarter, 2s. 8d.; per half-year, 5s. 3d.; per annum, 10s. 6d. Foreign:—Per annum, 15s. 2d., payable, in all cases, in advance.

**NOTICE.—Vol. XXXI. of "The Hospital" (October 1901, to March 1902), in handsome cloth binding is now on sale at the office of this Journal, price, 7s. 6d. Cases for binding the Numbers contained in this Volume 1s. 6d. Index to Vol. XXXI., 2½d., post free.**
**The Monthly Part for June will be ready on June 2nd. Price 6d., by post 8d.**

### Scraps and Gleanings.

As a memorial to Queen Victoria, a recreation ground has just been opened at Finchley. A sum of £9,500 has been spent on buying and laying out about 18 acres of land, a large part of which is to be reserved for cricket and football; £3,000 was raised privately, and the District Council themselves took the matter in hand, with a view to the provision of the remaining sum required.

At a recent meeting held at Reading and convened by the Mayor of Reading, it was unanimously resolved (1) that the workpeople of Reading should be invited to organise a scheme for helping the Royal Berks Hospital by a system of regular contributions; and (2) that the Mayor be requested to call a public meeting at an early date to place before the public the pressing needs of the Royal Berks Hospital.

The ordinary income for last year of the St. Helen's Hospital was £2,174, and the ordinary expenditure £2,104, leaving a balance of £70, which, together with £604 brought forward, and £751, income from endowment, made a sum of £1,425 odd, which will be used for furnishing the proposed extension of the hospital, the plans for which have already been accepted. The extension will about double the number of beds, and provide additional administrative accommodanion.

### The Student of Medicine.

### Appointments.

**E. C. Bailey**, M.B., B.S.Durh., appointed Certifying Surgeon under the Factory Act for the Crewe District of Cheshire. **K. de R. Bell**, M.R.C.S., L.R.C.P.Lond., appointed Certifying Surgeon under the Factory Act for the Uppingham District of Rutland. **A. L. Black**, appointed House Surgeon to the Royal Victoria Hospital, Belfast. **Rowland Hill Coombs**, M.D.Durh., appointed Medical Officer for the Rural District of Bedford. **E. L. Crowther**, M.D.Aberd., appointed a member of the Central Board of Health, Tasmania. **J. Crow**, M.B., Ch.B.Glasg., appointed Medical Officer and Public Vaccinator for the Largs and Fairlie Districts Parish Council. **Norman Davidson**, M.B., Ch.B.Aberd., appointed Medical Officer of Health for the Burgh of Peterhead. **Edmund Genny**, L.R.C.P.I., L.R.C.S.I., appointed Senior House Surgeon to Jervis Street Hospital, Dublin. **A. H. Grace**, L.S.A., appointed Certifying Surgeon under the Factory Act for the Chipping Sodbury District of Gloucestershire. **N. Bishop Harman**, M.A., M.B.Cantab., F.R.C.S.Eng., appointed Demonstrator of Pathology in the Middlesex Hospital Medical School. **H. Horrocks**, M.D.Lond., appointed Assistant Gynæcologist to the Perth Public Hospital, Western Australia. **O. C. Jones**, M.B.Oxon., appointed Certifying Surgeon under the Factory Act for the Ilfracombe District of Devon. **H. T. Kelsall**, M.D.Lond., appointed Ophthalmic Surgeon to the Perth Public Hospital, Western Australia. **J. C. Michell**, M.R.C.S., L.S.A., appointed Certifying Surgeon under the Factory Act for the Lynton District of Devonshire. **Ernest Arthur Milner**, M.B., C.M.Edin., appointed Medical Officer of Health for Lostwithiel. **J. M. Randle**, L.R.C.P.Lond., M.R.C.S., appointed Deputy Medical Officer of Health for the Borough of Helston, Cornwall, during the illness of the Medical Officer. **R. W. Richards**, M.D.Lond., appointed Certifying Surgeon under the Factory Act for the Dolgelly District of the county of Merioneth. **W. P. Richardson**, M.B., M.S.Edin., appointed Certifying Surgeon under the Factory Act for the Blisworth District of Northamptonshire. **John P. Ryan**, L.R.C.P.I., L.R.C.S.I., appointed Junior House Surgeon to Jervis Street Hospital, Dublin. **Cecil E. Shaw**, M.D., M.Ch.R.U.I., appointed Consulting Ophthalmic Surgeon to the County Antrim Infirmary, Lisburn. **James Shaw**, M.D.Glasg., appointed Certifying Surgeon under the Factory Act for the Aylesbury District of Bucks. **Howard Stevenson**, M.B., B.Ch., B.A.O.R.U.I., appointed House Surgeon to the Royal Victoria Hospital, Belfast. **F. Totman**, M.D.Lond., appointed Senior Surgeon to the Perth Public Hospital, Western Australia. **W. Trethowan**, M.B.Aberd., appointed Assistant Surgeon to the Perth Public Hospital, Western Australia. **Francis Tyndall**, M.R.C.S., L.R.C.P.Lond., appointed Certifying Surgeon under the Factory Act for the Westhoughton District of Lancashire. **Chisholm Williams**, F.R.C.S.Edin., appointed in charge of the Electro-Therapeutic Department, West London Hospital.

**"The Hospital" Institutional Library.**

"Hospitals and Asylums of the World." 4 Vols., with a Portfolio of Plans, complete £12 12s.
"Burdett's Hospitals and Charities, 1902." 5s.
"Cottage Hospitals, General, Fever, and Convalescent." 10s. 6d.
"Hospital Expenditure: The Commissariat." 2s. 6d.
"A Legal Handbook for the Use of Hospital Authorities." 2s. 6d.
"The Cottage Hospital Case Book or Register of Patients." 10s. and 12s. 6d.
"The Furnishing and Appliances of a Cottage Hospital." 6d.
All these are published by the Scientific Press, Ltd., and may be obtained through any bookseller, or direct from the publishers, 28 and 29 Southampton Street, Strand, London, W.C.

**An ATLAS of BACTERIOLOGY.**

Containing 111 Original Photo-Micrographs, with Explanatory Text. By CHARLES SLATER, M.A., M.B., M.R.C.S. (Eng.), F.C.S., and EDMUND J. SPITTA, L.R.C.P. (Lond.), M.R.C.S. (Eng.). Demy 8vo., cloth gilt, 7s. 6d. net.

"It is impossible to deny that this work fills a blank in the life of the student of bacteriology. The photographs are excellent and the letterpress, linking together and explaining the teaching of the illustrations, is clear, concise, and accurate."—*Nature.*

The Scientific Press, Ltd., 28 & 29 Southampton Street, Strand, London, W.C.

